# Real-world outcomes of ponatinib in heavily pretreated patients with chronic myeloid leukemia and Philadelphia chromosome-positive acute lymphoblastic leukemia

**DOI:** 10.1007/s00277-026-07053-6

**Published:** 2026-05-22

**Authors:** Seok Lee, Hyeoung-Joon Kim, June-Won Cheong, Ho-Jin Shin, Jee Hyun Kong, Sang Kyun Sohn, Sung-Soo Yoon, Dae Young Zang, Chul Won Jung, Jeong-A Kim, Sung-Eun Lee, Won Sik Lee, Yunsuk Choi, Inho Kim, Jae Joon Han, Min Kyoung Kim, So Young Chong, Da Jung Kim, Deog-Yeon Jo, Hawk Kim, Jae-Yong Kwak, Je-Hwan Lee, Jieun Uhm, Sewon Lee, Min-Yi Lee, Dong-Wook Kim

**Affiliations:** 1 Department of Hematology, Ewha Womans University Mokdong Hospital, Seoul, Republic of Korea; 2 Hematology-Oncology, Chonnam National University Hwasun Hospital, Hwasun, Jeollanam-do Republic of Korea; 3 Division of Hematology, Severance Hospital, Yonsei University College of Medicine, Seoul, Republic of Korea; 4 Division of Hematology-Oncology, Department of Internal Medicine, Pusan National University Hospital, Busan, Republic of Korea; 5 Division of Oncology and Hematology, Department of Internal Medicine, Wonju Severance Christian Hospital, Wonju, Republic of Korea; 6 Department of Hematology/Oncology, Kyungpook National University Hospital, Daegu, Republic of Korea; 7 Department of Internal Medicine, Seoul National University Hospital, Seoul, Republic of Korea; 8 Division of Hematology-Oncology, Department of Internal Medicine, Hallym University Sacred Heart Hospital, Anyang-si, Republic of Korea; 9 Division of Hematology-Oncology, Department of Medicine, Samsung Medical Center, Sungkyunkwan University School of Medicine, Seoul, Republic of Korea; 10 Department of Internal Medicine, St. Vincent’s Hospital, College of Medicine, The Catholic University of Korea, Seoul, South Korea; 11 Department of Hematology, Seoul St. Mary’s Hospital, College of Medicine, The Catholic University of Korea, Seoul, Republic of Korea; 12 Department of Hematology and Oncology, Internal Medicine, Busan Paik Hospital, Inje University College of Medicine, Busan, Republic of Korea; 13 Department of Hematology, Asan Medical Center, University of Ulsan College of Medicine, Seoul, Republic of Korea; 14 Department of Hematology and Medical Oncology, Kyung Hee University College of Medicine, Seoul, Republic of Korea; 15 Division of Hemato-Oncology, Department of Internal Medicine, Yeungnam University College of Medicine, Daegu, Republic of Korea; 16 Department of Internal Medicine, CHA Bundang Medical Center, CHA University School of Medicine, Seongnam, Republic of Korea; 17 Division of Hematology/Oncology, Department of Internal Medicine, Kosin University College of Medicine, Kosin University Gospel Hospital, Busan, Republic of Korea; 18 Division of Hematology and Oncology, Department of Internal Medicine, Chungnam National University Hospital, Daejeon, Republic of Korea; 19 Division of Hematology, Gachon University Gil Medical Center, Gachon University College of Medicine, Incheon, Republic of Korea; 20 Department of Internal Medicine, Jeonbuk National University Medical School, Jeonju, Republic of Korea; 21 Division of Hematology and Oncology, Department of Internal Medicine, Hanyang University College of Medicine, Hanyang University Seoul Hospital, Seoul, Republic of Korea; 22 Korea Otsuka Pharmaceutical Co., Ltd, Seoul, Republic of Korea; 23 Department of Hematology, Uijeongbu Eulji Medical Center, Uijeongbu, Republic of Korea; 24 Leukemia Omics Research Institute, Uijeongbu Eulji Medical Center, Eulji University Uijeongbu Campus, 712 Dongil-ro, Uijeongbu, 11759 Korea

**Keywords:** Ponatinib, Post-marketing surveillance, Chronic myeloid leukemia, Acute lymphoblastic leukemia

## Abstract

To evaluate the real-world efficacy and safety of ponatinib, we conducted a multicenter, prospective post-marketing surveillance study in patients with chronic phase (CP), accelerated phase (AP), or blast phase (BP) chronic myeloid leukemia (CML) or Philadelphia chromosome–positive acute lymphoblastic leukemia (Ph + ALL), who were resistant or intolerant to prior tyrosine kinase inhibitors (TKIs) or harbored the T315I mutation. A total of 148 patients were enrolled across 24 sites: 115 with CML (92 CP-CML, 12 AP-CML, 11 BP-CML) and 33 with Ph + ALL. Ponatinib was administered as third-line therapy in 70 patients (47.3%), and 127 (85.8%) initiated treatment at 45 mg once daily. Sixty-three patients (42.6%) began ponatinib due to resistance or intolerance to prior TKIs. In CP-CML, the overall major molecular response (MMR) rate was 43.2% (35/81), with a cumulative MMR incidence of 23.9% at week 24 among patients without prior MMR. In Ph + ALL, the overall MMR and complete cytogenetic response (CCyR) rates were 82.4% (14/17) and 75.0% (3/4), respectively. CP-CML patients receiving ponatinib as earlier-line therapy achieved higher MR4.5 rates than those treated later (47.4% vs. 16.1%, *p* = 0.011). The most common adverse drug reactions were rash (21.6%, 32/148), hypertension (12.2%, 18/148), and pyrexia (10.1%, 15/148); two vascular occlusive events occurred. Ponatinib showed favorable real-world efficacy and manageable safety in Korean patients. Early, high-dose therapy suggests a trend toward deep molecular responses, supporting its clinical benefit in real-world practice. (ClinicalTrials.gov Identifier: NCT03709017; registered October 15, 2018)

**Supplementary Information:**

The online version contains supplementary material available at https://doi.org/10.1007/s00277-026-07053-6.

## Introduction

Chronic myeloid leukemia (CML) and Philadelphia chromosome-positive acute lymphoblastic leukemia (Ph + ALL) are hematologic malignancies characterized by the *BCR::ABL1* fusion gene, resulting from the translocation t(9;22)(q34;q11) [1, 2]. The constitutively active *BCR::ABL1* tyrosine kinase drives leukemogenesis and serves as a critical therapeutic target. The advent of tyrosine kinase inhibitors (TKIs), including imatinib and second-generation agents such as dasatinib, radotinib, bosutinib, and nilotinib, has dramatically improved outcomes in CML and Ph + ALL.

Despite this progress, resistance or intolerance to prior TKIs remains a major challenge. Mutations within the *ABL1* kinase domain (KD)—especially T315I—confer resistance to most first- and second-generation TKIs [1, 3].

Ponatinib is a third-generation TKI specifically designed to inhibit most *ABL1* KD mutations, including T315I [4]. In the pivotal phase 2 Ponatinib Ph + ALL and CML Evaluation (PACE) trial, ponatinib produced substantial responses in resistant or intolerant CML or Ph + ALL [5], leading to approval in multiple countries, including Korea on June 26, 2017, for patients harboring T315I or with TKI resistance/intolerance. More recently, the phase 3 PhALLCON study established ponatinib in combination with chemotherapy as first-line therapy for newly diagnosed Ph + ALL [6].

Although ponatinib’s efficacy has been proven in trials, real-world data—particularly in Asian populations—remain limited. A Japanese post-marketing surveillance (PMS) study reported outcomes that differed from previous international clinical studies in terms of target diseases, treatment timing, and dosage [7]. Furthermore, chronic TKI exposure can lead to long-term adverse events (AEs) such as nephropathy and arterial occlusive events (AOEs). Although strategies to balance efficacy and safety through dose adjustment have been discussed in the literature, real-world evidence describing ponatinib use in Asian clinical practice—including dosing patterns and associated safety outcomes—remains limited [8–12]. To bridge this gap, we conducted a 6-year study in Korea following ponatinib approval, in accordance with local risk management plan (RMP) requirements. The study evaluated real-world safety, including vascular occlusive events (VOEs), and efficacy of ponatinib in Korean patients with chronic phase (CP), accelerated phase (AP), or blast phase (BP) CML and Ph + ALL in real world clinical settings.

## Methods

### Study design and population

This PMS study assessed the real-world safety and efficacy of ponatinib in patients with CML or Ph + ALL in the Republic of Korea. Conducted from June 26, 2017, to June 25, 2023, across 24 institutions, all patients who received ponatinib during the first 2 years after its launch were enrolled, and enrollment continued over the subsequent 4 years to include all eligible patients. Each patient was monitored from baseline (prior to ponatinib initiation) for at least 3 months, with more than 10% followed for up to 6 months. Assessments were performed under routine clinical practice conditions at baseline and at 3 and 6 months.

Eligible patients had CP, AP, or BP CML or Ph + ALL (1) resistant or intolerant to prior TKIs, or (2) harboring the T315I mutation. Patients were excluded if they had a known or suspected hypersensitivity to ponatinib or any of its excipients, had genetic disorders such as galactose intolerance, Lapp lactase deficiency, or glucose-galactose malabsorption, or were otherwise considered unsuitable for participation based on the investigator’s clinical judgment. Following informed consent, ponatinib-naïve patients were enrolled in the study.

### Data collection

Collected data included demographics, comorbidities, mutation status (including T315I), prior TKI history, ponatinib dosing, and safety and efficacy outcomes. Safety evaluations encompassed all laboratory and clinical AEs occurring from the first dose until 14 days after the last dose. All AEs reported during this period were documented and analyzed.

Serious adverse events (SAEs) were defined as any AE occurring at any dose that: (1) resulted in death; (2) was life-threatening; (3) required inpatient hospitalization or prolonged existing hospitalization; (4) resulted in persistent or significant disability or incapacity; (5) was a congenital anomaly or birth defect; or (6) was medically significant. All AEs were coded using the Medical Dictionary for Regulatory Activities (MedDRA) version 25.0 and graded according to the Common Terminology Criteria for Adverse Events (CTCAE) version 5.0. VOEs comprised AOEs (coronary, cerebral, peripheral, and retinal artery occlusions) and venous thrombotic/embolic events (VTEs), including retinal vein thrombosis and vision loss.

Efficacy was evaluated according to the 2020 European LeukemiaNet (ELN) criteria. Although the study was initiated under the 2013 guidelines, the collected response data were classified according to the 2020 ELN definitions to ensure alignment with current clinical standards [13, 14]. Hematologic response was defined as complete hematologic response (CHR). Cytogenetic response was based on at least 20 metaphase cells, with the proportion of Ph+ metaphase cells determining response. Major cytogenetic response (MCyR) included both complete cytogenetic response (CCyR; no detectable Ph+ metaphase cells) and partial cytogenetic response (PCyR; 1–35% Ph+ metaphase cells). Molecular response by quantitative reverse transcription polymerase chain reaction (qRT-PCR) on the International Scale (IS) was classified as MR1.0 (*BCR::ABL1* ≤ 10%), MR2.0 (*BCR::ABL1* ≤ 1%), major molecular response (MMR/MR3.0; *BCR::ABL1* ≤ 0.1%), and MR4.5 (*BCR::ABL1* ≤ 0.0032%). Throughout the study, cytogenetic and molecular responses were collected and analyzed separately.

### Statistical analysis

Analysis was performed using SAS^®^ version 9.4 64-bit or higher (SAS Institute Inc., Cary, NC, USA) and R version 4.4.3. Continuous variables were summarized with the number of subjects, mean, standard deviation, median, minimum, and maximum values. Categorical variables were presented as frequencies and percentages.

Safety was evaluated by the number and proportion of patients experiencing AEs, adverse drug reactions (ADRs), SAEs, and serious adverse drug reactions (SADRs), along with the total number of events. Efficacy was assessed by treatment response rates, reported as the number and proportion of patients achieving a response with exact 95% confidence intervals (CIs). Response data collected more than 28 days after ponatinib discontinuation were excluded. Overall response was defined as the best response achieved at any time during treatment to reflect real‑world clinical practice, where assessment intervals and response evaluation timings vary across institutions. Cumulative incidence of response was analyzed using the cumulative incidence function (CIF) among patients without prior response and with available data during treatment. Response by timepoint was defined as the proportion of patients achieving a response by specific timepoints (e.g., week 12, week 18, week 24, week 30), regardless of prior response status.

## Results

### Patient characteristics

A total of 148 patients were enrolled, with data collected via electronic case report forms (eCRFs). Safety was evaluated in all 148 patients who received at least one dose of ponatinib and completed follow-up. Among these, 115 patients had CML (92 CP-CML, 12 AP-CML, 11 BP-CML), and 33 patients had Ph + ALL. Efficacy was assessed in an efficacy-evaluable population of 131 patients with available hematologic, cytogenetic, or molecular response data following ponatinib administration (107 CML, including 86 CP-CML; 24 Ph + ALL). The remaining 17 patients were excluded from the efficacy analysis because they lacked evaluable post-treatment response data.

The mean time from diagnosis to ponatinib initiation was 65.7 months. Regarding prior TKI treatment history, 42.6% (63/148) had experienced both resistance and intolerance, 35.1% (52/148) had experienced resistance only, and 21.6% (32/148) had experienced intolerance only (Table 1). One patient was T315I-positive without prior TKI exposure. A total of 109 patients (73.6%) had at least one comorbidity, including hypertension (23.0%, 34/148), diabetes mellitus (18.2%, 27/148), history of ischemic disease (6.8%, 10/148), dyslipidemia (2.7%, 4/148), and venous thromboembolism (0.7%, 1/148).

**Table 1:** Baseline characteristics of patients

	Overall (*N* = 148)	All Types of CML (*N* = 115)	CP-CML (*N* = 92)	Ph + ALL (*N* = 33)
Age^a^	56.0 (19.0 ~ 81.0)	57.0 (19.0 ~ 81.0)	57.5 (19.0 ~ 81.0)	54.0 (21.0 ~ 81.0)
BMI (kg/m²)	22.8 (16.4 ~ 57.5)	23.5 (16.4 ~ 57.5)	24.3 (18.8 ~ 57.5)	21.3 (17.5 ~ 35.7)
Body Weight (kg)	65.0 (41.0 ~ 201.0)	66.6 (48.6 ~ 201.0)	67.5 (54.0 ~ 201.0)	58.8 (41.0 ~ 97.1)
Treatment Disease				
CP-CML	92 (62.2)			
AP-CML	12 (8.1)			
BP-CML	11 (7.4)			
Ph + ALL	33 (22.3)			
Comorbidities				
Hypertension	34 (23.0)	25 (21.7)	21 (22.8)	9 (27.3)
Diabetes	27 (18.2)	15 (13.0)	12 (13.0)	12 (36.4)
Dyslipidemia	4 (2.7)	2 (1.7)	2 (2.2)	2 (6.1)
History of Ischemic Disease	10 (6.8)	8 (7.0)	6 (6.5)	2 (6.1)
Venous Thromboembolism	1 (0.7)	0	0	1 (3.0)
*BCR::ABL1* Mutation				
Collected	86 (58.1)	72 (62.6)	52 (56.5)	14 (42.4)
Positive	48 (32.4)	36 (31.3)	20 (21.7)	12 (36.4)
T315I Mutation	25 (16.9)	19 (16.5)	8 (8.7)	6 (18.2)
Negative	38 (25.7)	36 (31.3)	32 (34.8)	2 (6.1)
Prior TKI Treatment, n				
Treatment-naïve	1	0	0	1
Previously treated	147	115	92	32
Treatment Line				
1 st line	1 (0.7)	0	0	1 (3.0)
2nd line	34 (23.0)	28 (24.4)	20 (21.7)	6 (18.2)
3rd line	70 (47.3)	46 (40.0)	34 (37.0)	24 (72.7)
4th line	32 (21.6)	30 (26.1)	27 (29.4)	2 (6.1)
5th line	10 (6.8)	10 (8.7)	10 (10.9)	0
6th line	1 (0.7)	1 (0.9)	1 (1.1)	0
Result of Prior TKIs Treatment				
Resistance to TKIs	52 (35.1)	35 (30.4)	23 (25.0)	17 (51.5)
Intolerance to TKIs	32 (21.6)	24 (20.9)	20 (21.7)	8 (24.2)
Both Resistance and Intolerance	63 (42.6)	56 (48.7)	49 (53.3)	7 (21.2)
Diagnosis-to-Treatment Interval (month)^b^, mean	65.7	75.4	82.0	31.7
Treatment Duration (day)^c^	169.5 (8.0 ~ 403.0)	171.0 (8.0 ~ 330.0)	175.0 (8.0 ~ 330.0)	129.0 (14.0 ~ 403.0)
Initial Daily Dose^d^				
45 mg/day	127 (85.8)	107 (93.0)	86 (93.5)	20 (60.6)
30 mg/day	17 (11.5)	5 (4.4)	3 (3.3)	12 (36.4)
15 mg/day	4 (2.7)	3 (2.6)	3 (3.3)	1 (3.0)
Average Daily Dose over 6-months (mg/day)^e^	43.4 (15.0 ~ 45.0)	44.4 (15.0 ~ 45.0)	41.6 (15.0 ~ 45.0)	35.3 (15.0 ~ 45.0)
Average Daily Dose (mg/day)^f^	42.6 (15.0 ~ 45.0)	44.0 (15.0 ~ 45.0)	40.0 (15.0 ~ 45.0)	34.8 (15.0 ~ 45.0)

Values are given as n (%) or median (range), unless otherwise specified.

*CML *chronic myeloid leukemia, *CP *chronic phase, *AP *accelerated phase, *BP *blast phase, *Ph + ALL *Philadelphia chromosome-positive acute lymphoblastic leukemia, *TKI* tyrosine kinase inhibitor

(a) Age is defined as the patient’s age at the time of baseline assessment, (b) Diagnosis-to-treatment interval is defined as the period between the initial diagnosis of leukemia and the initiation of ponatinib therapy; one month is defined as 28 days, (c) Treatment duration is defined as the total period from the treatment start date to the treatment end date, including any drug-free periods in between, (d) Initial daily dose is defined as the dose administered on the first day of treatment, (e) Average daily dose over 6-months is defined as the total cumulative dose administered divided by the total number of treatment days within the 6-month period, including drug-free periods, (f) Average daily dose is defined as total dose administered divided by the total number of treatment days, excluding drug-free periods

Baseline *BCR::ABL1* mutation testing was performed in 58.1% (86/148), with 32.4% (48/148) positive. The T315I mutation was detected in 16.9% (25/148) of patients (Table 1).

### Ponatinib administration

The median treatment duration was 169.5 days, with a median daily dose of 42.6 mg. Most patients initiated ponatinib at 45 mg/day (85.8%, 127/148), including 93.0% (107/115) of all CML patients and 93.5% (86/92) of CP-CML patients, compared with 60.6% (20/33) of Ph + ALL patients. Ponatinib was most frequently administered as third-line therapy, occurring in 47.3% (70/148) of all patients, 40.0% (46/115) of CML patients, 37.0% (34/92) of CP-CML patients, and 72.7% (24/33) of Ph + ALL patients (Table 1).

### Efficacy in patients with CP-CML

**Fig. 1:**
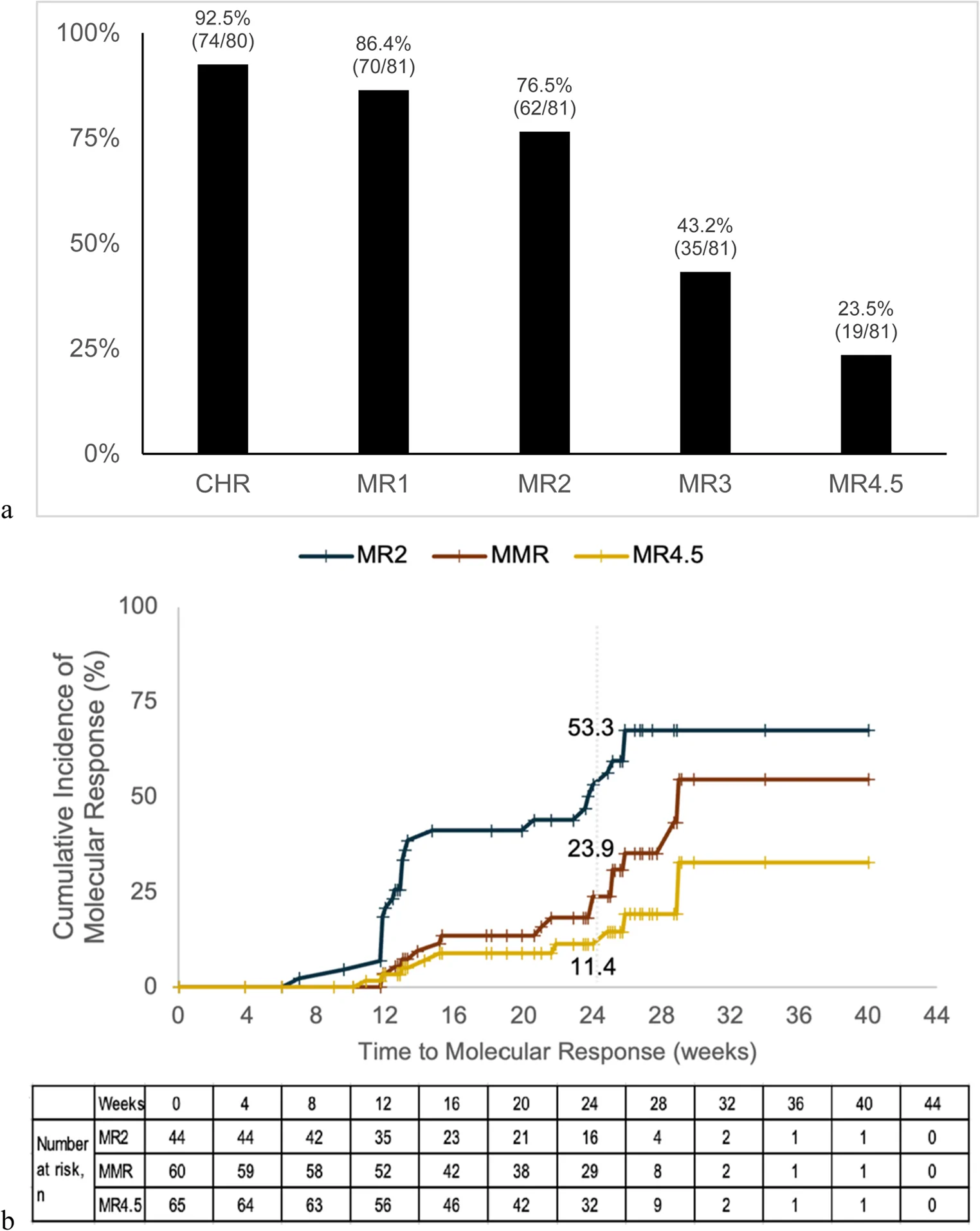
Efficacy of ponatinib in patients with CP-CML (a) Overall response in patients with CP-CML (b) Cumulative incidence of MR2, MMR and MR4.5 in patients with CP-CML. The table shows the number at risk at each time point; patients who did not achieve a response were censored at their last molecular assessment

**Fig. 2:**
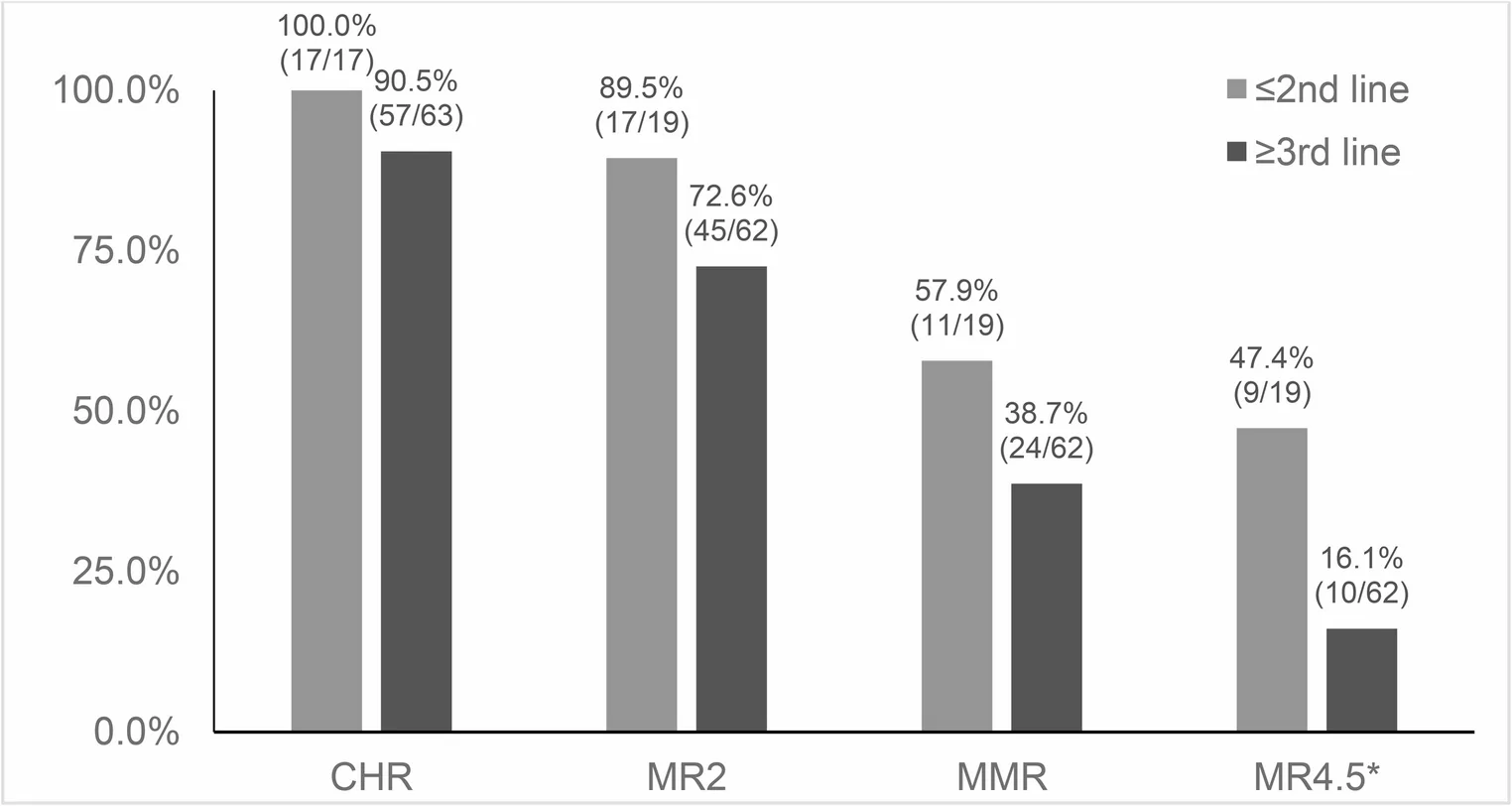
Overall response according to ponatinib treatment line in patients with CP-CML, *p-value < 0.05 for MR4.5 in second-line or earlier treatment compared with MR4.5 in third-line or later treatment

**Fig. 3:**
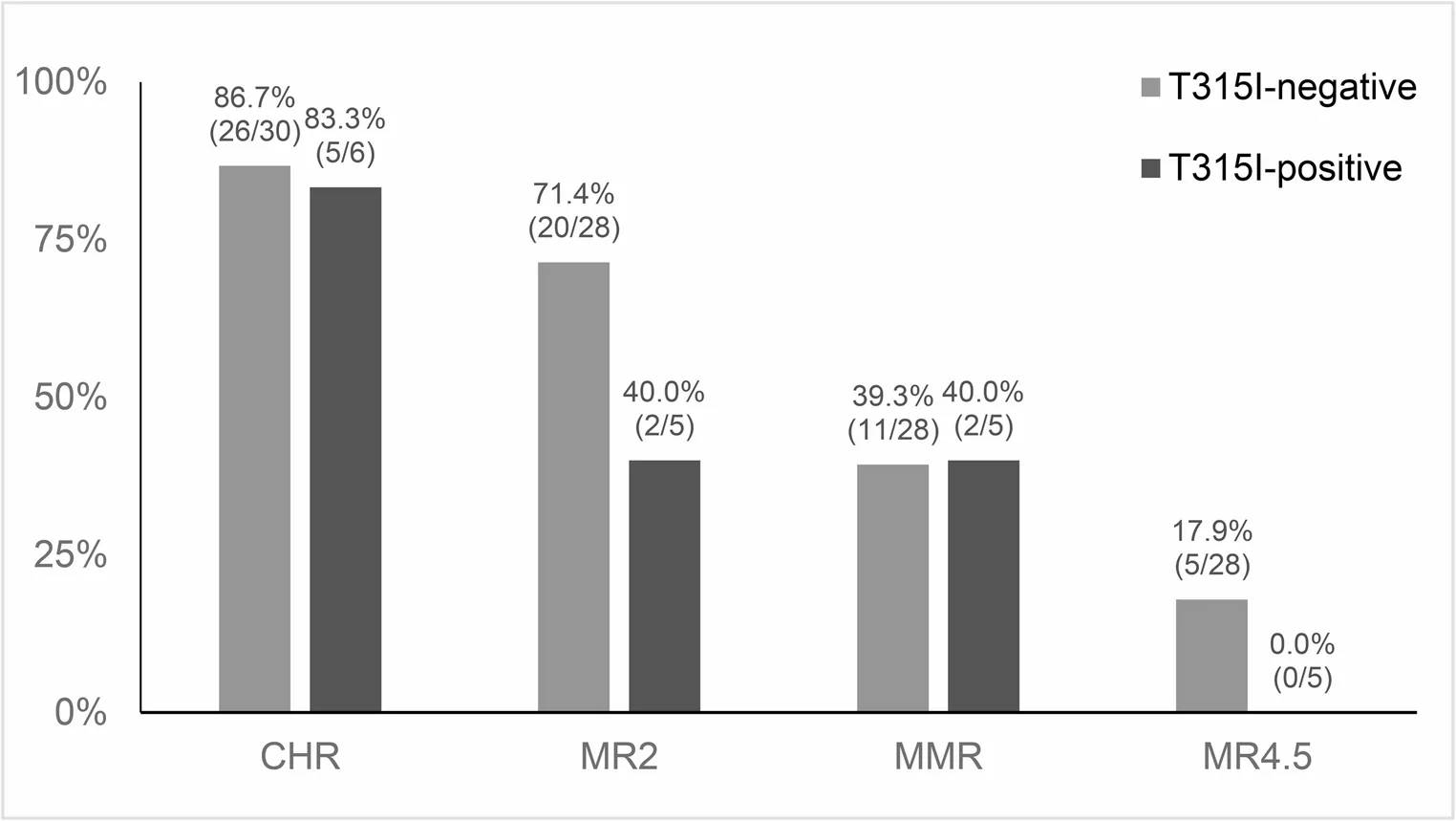
Overall response according to mutation T315I status in patients with CP-CML

In CP-CML, the overall rates were: CHR 92.5% (74/80), MCyR 75.0% (21/28), CCyR 60.7% (17/28), MR2 76.5% (62/81), MMR 43.2% (35/81), and MR4.5 23.5% (19/81) (Fig. 1a). The cumulative incidence by week 24 was 53.3% for MR2, 23.9% for MMR, and 11.4% for MR4.5 (Fig. 1b). Overall response rates were higher with early use (second-line or earlier)—including CHR 100.0% (17/17), MR2 89.5% (17/19), MMR 57.9% (11/19), and MR4.5 47.4% (9/19)—compared with third-line or later use (CHR 90.5% (57/63), MR2 72.6% (45/62), MMR 38.7% (24/62), and MR4.5 16.1% (10/62)), with a significant difference observed for MR4.5 (*p* = 0.011) (Fig. 2). By timepoint, MMR increased progressively: week 12, 25.7% (9/35); week 18, 29.4% (20/68); week 24, 36.0% (27/75); and week 30, 43.2% (35/81) (Supplementary Table 1). In T315I-positive patients, overall rates for CHR, MR2, and MMR were 83.3% (5/6), 40.0% (2/5), and 40.0% (2/5), respectively, with no MR4.5 achieved (0/5). T315I-negative patients achieved overall rates of CHR 86.7% (26/30), MR2 71.4% (20/28), MMR 39.3% (11/28), and MR4.5 17.9% (5/28) (Fig. 3). No statistically significant differences in response rates were observed by mutation status.

### Efficacy in patients with all types of CML

**Fig. 4:**
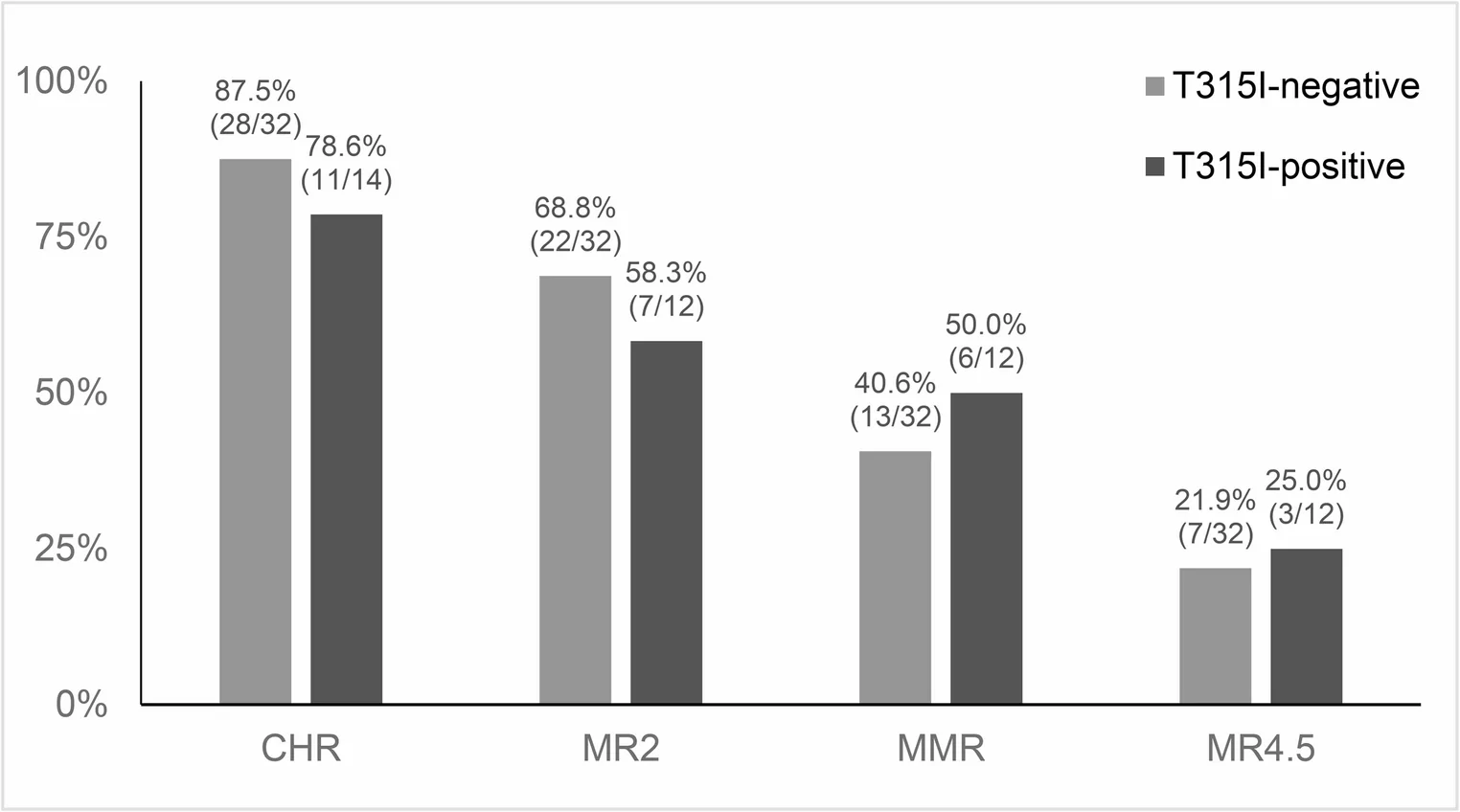
Overall response according to mutation T315I status in patients with all types of CML

Among all evaluable CML patients, overall response rates were as follows: CHR 90.6% (87/96), MCyR 69.4% (25/36), CCyR 58.3% (21/36), MR2 74.0% (74/100), MMR 46.0% (46/100), and MR4.5 27.0% (27/100). In T315I-positive patients, overall rates were: CHR 78.6% (11/14), MR2 58.3% (7/12), MMR 50.0% (6/12), and MR4.5 25.0% (3/12). T315I-negative patients achieved overall rates of CHR 87.5% (28/32), MR2 68.8% (22/32), MMR 40.6% (13/32), and MR4.5 21.9% (7/32) (Fig. 4). No statistically significant differences in response rates were observed by mutation status. Overall response rates were higher with second-line or earlier use (CHR 95.5%; MR2 80.8%; MMR 57.7%; MR4.5 46.2%) than with third-line or later use (CHR 89.2%; MR2 71.6%; MMR 41.9%; MR4.5 20.3%). MMR increased over time: week 12, 31.8% (14/44); week 18, 35.3% (30/85); week 24, 40.2% (37/92); and week 30, 45.0% (45/100).

### Efficacy in patients with Ph + ALL

In Ph + ALL, patients with available cytogenetic data (4/4) achieved overall rates of 100% (4/4) for MCyR and 75.0% (3/4) for CCyR. Overall molecular response rates were 82.4% (14/17) for MMR and 64.7% (11/17) for MR4.5; MMR increased from 72.7% by week 12 to 82.4% by week 24, remaining stable through week 30.

### Adverse drug reactions

A total of 207 ADRs occurred in 57.4% (85/148) of patients, with the most common being rash (21.6%, 32/148), hypertension (12.2%, 18/148), and pyrexia (10.1%, 15/148) (Table 2). SAEs were reported in 18.9% (28/148) of patients (53 events), and SADRs in 8.8% (13/148; 24 events) (Supplementary Table 2).

**Table 2:** Summary of adverse drug reactions

Adverse drug reactions in ≥ 3.0%	Overall (*N* = 148)	
	Incidence *n* (%)	Event case
Total	85 (57.4)	[207]
Rash	32 (21.6)	[36]
Hypertension^a^	18 (12.2)	[18]
Pyrexia	15 (10.1)	[16]
Neutropenia	7 (4.7)	[7]
Platelet count decreased	6 (4.1)	[8]
Thrombocytopenia	6 (4.1)	[7]
Headache	6 (4.1)	[7]
Aspartate aminotransferase increased	6 (4.1)	[6]
Febrile neutropenia	5 (3.4)	[8]
Neutrophil count decreased	5 (3.4)	[6]
Alanine aminotransferase increased	5 (3.4)	[5]
Myalgia	5 (3.4)	[5]

MedDRA version 25.0

ADRs were analyzed based on investigator-reported terms, not laboratory test criteria.

a. Among the patients, 3 had worsening hypertension at baseline, and 15 developed new-onset ADRs during treatment.

Two VOEs occurred in 1.4% (2/148) of patients, including one AOE in 0.7% (1/148). Neither was classified as an SAE or resulted in death, and both were considered related to pre-existing conditions. One case involved a 46-year-old male with BP-CML, diagnosed in January 2018, who underwent stem cell transplantation later that year and subsequently developed chronic graft-versus-host disease (GVHD) in January 2019. Ponatinib was initiated in March 2020 at 45 mg/day. Two weeks later, epigastric tenderness led to a dose reduction to 30 mg/day. Eleven weeks after initiation, right arm swelling occurred, and peripheral arterial occlusive disease (PAOD) was suspected. The dose was further reduced to 15 mg/day, resulting in symptom resolution; ponatinib was later re-escalated without recurrence. The investigator attributed the event to both ponatinib and the underlying condition. The second case involved a 37-year-old male with BP-CML diagnosed in 2009 and coronary artery disease diagnosed in 2021. After starting ponatinib 45 mg/day in October 2021, he developed chest pain approximately 4 months later. The event was assessed as an exacerbation of pre-existing coronary artery disease and was deemed unrelated to ponatinib.

Two deaths occurred, both unrelated to ponatinib and attributed to underlying conditions. One case involved an 81-year-old female with CP-CML diagnosed in June 2001, who had previously received imatinib, bosutinib, nilotinib, and dasatinib. Her medical history included diabetes mellitus, hypertension, and stage 3 chronic kidney disease (CKD). Ponatinib was initiated in December 2018 following dasatinib intolerance. Fourteen days after initiation, she developed acute renal failure on CKD. Despite discontinuation of ponatinib and administration of diuretics, antibiotics, and arrhythmia control, she progressed to respiratory failure and died. The second case involved a 61-year-old male with Ph + ALL diagnosed in 2020, who received ponatinib as third-line therapy following more than one year of imatinib and dasatinib. After approximately two months of treatment, he experienced disease progression of B-lymphoblastic leukemia/lymphoma and died during hospitalization.

## Discussion

This PMS study collected real-world data from 148 patients treated with ponatinib across 24 institutions in the Republic of Korea between June 26, 2017, and June 25, 2023. Most patients had CP-CML (62.2%), followed by Ph + ALL (22.3%). Regarding treatment patterns, 76.4% received ponatinib as a third-line treatment or later, and 85.8% started at 45 mg/day (93.5% in CP-CML, 60.6% in Ph + ALL). These findings indicate that ponatinib is predominantly used for advanced, heavily pretreated patients in Korea.

Compared with a Japanese PMS study of 724 patients conducted from November 2016 to June 2018, the Korean cohort showed markedly different treatment patterns. In Japan, only 26.7% of patients had CP-CML and 53.9% had Ph + ALL, whereas ponatinib was more frequently used as second-line therapy (48.5%), with 51.2% of patients starting at 15 mg/day [7]. In contrast, Korean patients more often received ponatinib in later lines, at higher starting doses (45 mg/day in 85.8%), and for a smaller proportion of Ph + ALL cases. The proportion of Korean CP-CML patients with both TKI intolerance and resistance (53.3%, 49/92) was also higher than the combined intolerance group (intolerance only plus intolerance and resistance) in Japan (32.1%, 62/193) [7]. These variations reflect differing reimbursement policies. In Japan, PMDA approval ensures broad insurance coverage for all indications, enabling earlier access [15]. Conversely, Korea’s HIRA system restricts reimbursement to specific treatment lines based on demonstrated clinical and economic value [16, 17]. Consequently, ponatinib reimbursement is restricted to second-line or later therapy in CML and third-line in Ph + ALL, explaining its later and more selective use in Korea.

Among CP-CML patients, the cumulative MMR of 54.6% at week 30 (Fig. 1b) in this Korean PMS study compares favorably with the 58.8% MMR at week 52 in the Japanese PMS study [7], suggesting consistent real-world efficacy despite differing follow-up schedules, dosing, and patient characteristics. MMR (43.2%) and MCyR (75.0%) rates exceeded those of the phase 2 PACE trial (MMR 34% and MCyR 56%) [5] and were comparable or superior to those in the OPTIC trial (MMR 34.4%, MCyR 50.5% at 45 mg/day; MMR 24.7% and 23.1%, MCyR 33.3% and 43.8% at 30 and 15 mg/day) [18]. Although cross-study comparisons are limited, these findings support the clinical efficacy of ponatinib in real-world Korean patients.

The MR2 rate of 72.0% by week 24 in CP-CML patients was higher than the 6-month MR2 rate of 40.9% in the 45 mg cohort of the OPTIC trial [18] (Supplementary Table 1). The average daily dose by 6 months in this study (40 mg/day) was higher than OPTIC’s 35.2 mg/day. However, the Korean cohort’s lower body weight (67.5 kg) and BMI (24.3 kg/m²) compared with OPTIC’s predominantly Western population (78.1 kg, BMI 27.0 kg/m²; 17.0% Asian, 77.7% White) present a plausible mechanism for greater systemic exposure at comparable doses [18, 19]. Such physiological differences align with prior reports showing that Asian patients achieve similar or higher plasma TKI levels at lower doses due to smaller body surface area (BSA) [20, 21], potentially contributing to the robust responses observed.

In CP-CML, overall MMR rates were higher with second-line or earlier ponatinib use (57.9%) than with third-line or later treatment (38.7%), with MR4.5 rates also significantly higher (47.4% vs. 16.1%; *p* = 0.011). These findings suggest a trend toward deeper and faster molecular responses with earlier ponatinib use, consistent with the TOPASE study in France [22]. While these results suggest a reconsideration of guidelines to allow earlier ponatinib use, further prospective studies with extended follow-up are warranted to validate long-term durability. Even in later treatment lines, the MMR rate (38.7%) was comparable to the PACE trial (34%) [5], supporting ponatinib’s role as an effective option and a potential bridge to stem cell transplantation or other definitive therapies.

AOEs are a key safety concern with ponatinib. In this Korean PMS study, VOEs occurred in 1.4% of patients, including AOEs in 0.7%, substantially lower than previous reports: 25% in PACE, 9.6% in OPTIC (45 mg cohort) [18, 23], and 6.5% in the Japanese PMS study [7]. The lower incidence may reflect the study’s shorter follow-up, potentially underestimating time-dependent events [24] alongside the non-interventional design. This real-world setting permitted flexible, response-based dose adjustments, enabling clinicians to maintain efficacy while proactively mitigating vascular risk—a strategy consistent with dose-reduction approaches for other TKIs [8–12, 25].

Early-onset toxicities and baseline risk factors, particularly hypertension—a known predictor of vascular events—were monitored [26]. Baseline hypertension (23.0%) and ADR-related hypertension (12.2%) in this study were lower than in the PACE (53.5%; 32%) and OPTIC (31.0%; 37%) cohorts, likely contributing to the lower AOE rates observed [23, 27]. Compared to the Japanese PMS study (26.8% baseline hypertension, 7.7% ADR-related hypertension), the Korean cohort showed higher ADR-related hypertension but fewer AOEs. This likely reflects differences in dosing and duration; 85.8% of Korean patients started at 45 mg/day (average 42.6 mg/day) for a shorter period (169.5 days). In contrast, only 19.8% of Japanese patients started at 45 mg/day (15.3% receiving ≥ 35 mg/day on average), with longer treatment durations (258 days) [7]. These dosing differences, along with lower baseline hypertension may explain the varied safety profiles. Collectively, lower AOE incidences in Asian cohorts compared to Western datasets support potential ethnic differences in tolerability, indicating that ponatinib is safe for Asian populations when managed with proper monitoring.

The ADR profile was consistent with previous studies across ethnicities. Despite higher dose exposure in this cohort due to lower BMI and body weight, AE rates did not increase, and no unique toxicities were observed in Asian patients, suggesting potentially improved tolerability. Further long-term, comparative, and pharmacokinetic studies are needed to confirm these findings and guide dosing.

The relatively short follow-up duration (approximately 6 months) remains a key limitation. While early response rates are encouraging, this timeframe is insufficient to evaluate long-term outcomes such as progression-free survival (PFS), overall survival (OS), and duration of response (DOR). Given that vascular events are often cumulative, the possibility of an underestimation due to short follow-up necessitates tempering safety claims relative to historical datasets until extended monitoring data are available. Additionally, the non-interventional design may have limited consistent data capture compared with controlled trials. Further long-term studies are needed to confirm these findings and guide individualized dosing.

Nonetheless, favorable efficacy was observed during the relatively short follow-up period, particularly in patients with CP-CML when treatment was initiated in earlier lines. Across the entire cohort of patients with CML and Ph + ALL, ponatinib showed an acceptable safety profile in real-world clinical practice with no unexpected adverse events, even at higher starting doses. These findings suggest that with appropriate monitoring and dose management, ponatinib remains a safe and effective option for heavily pretreated patients, especially when intervention occurs early in the disease course.

## Supplementary Information

Below is the link to the electronic supplementary material.

Supplementary Material 1

## Data Availability

The data are not publicly available to protect study participant privacy.
