# Supplementary material for: Real-world outcomes of ponatinib in heavily pretreated patients with chronic myeloid leukemia and Philadelphia chromosome-positive acute lymphoblastic leukemia
Source: Ann Hematol. 2026 May 22;105(7):315. doi: 10.1007/s00277-026-07053-6 (PMC13331954; doi:10.1007/s00277-026-07053-6)
Supplement: Supplementary file 1 — Supplementary Material 1 [file 277_2026_7053_MOESM1_ESM.docx]

**Journal Name: Annals of Hematology**

**Article Title: Real-World Outcomes of Ponatinib in Heavily Pretreated Patients with Chronic Myeloid Leukemia and Philadelphia Chromosome-Positive Acute Lymphoblastic Leukemia**

**Authors:**

Seok Lee^1^, Hyeoung-Joon Kim^2^, June-Won Cheong^3^, Ho-Jin Shin ^4^, Jee Hyun Kong^5^, Sang Kyun Sohn^6^, Sung-Soo Yoon^7^, Dae Young Zang^8^, Chul Won Jung^9^, Jeong-A Kim^10^, Sung-Eun Lee^11^, Won Sik Lee^12^, Yunsuk Choi^13^, Inho Kim ^7^, Jae Joon Han^14^, Min Kyoung Kim^15^, So Young Chong^16^, Da Jung Kim^17^, Deog-Yeon Jo^18^, Hawk Kim^19^, Jae-Yong Kwak^20^, Je-Hwan Lee^13^, Jieun Uhm^21^, Sewon Lee^1^, Min-Yi Lee^22^, Dong-Wook Kim^23, 24^

1 Department of Hematology, Ewha Womans University Mokdong Hospital, Seoul, Republic of Korea

2 Hematology-Oncology, Chonnam National University Hwasun Hospital, Hwasun, Jeollanam-do, Republic of Korea

3 Division of Hematology, Severance Hospital, Yonsei University College of Medicine, Seoul, Republic of Korea

4 Division of Hematology-Oncology, Department of Internal Medicine, Pusan National University Hospital, Busan, Republic of Korea

5 Division of Oncology and Hematology, Department of Internal Medicine, Wonju Severance Christian Hospital, Wonju, Republic of Korea

6 Department of Hematology/Oncology, Kyungpook National University Hospital, Daegu, Republic of Korea

7 Department of Internal Medicine, Seoul National University Hospital, Seoul, Republic of Korea

8 Division of Hematology-Oncology, Department of Internal Medicine, Hallym University Sacred Heart Hospital, Anyang-si, Republic of Korea

9 Division of Hematology-Oncology, Department of Medicine, Samsung Medical Center, Sungkyunkwan University School of Medicine, Seoul, Republic of Korea

10 Department of Internal Medicine, St. Vincent's Hospital, College of Medicine, The Catholic University of Korea, Seoul, South Korea

11 Department of Hematology, Seoul St. Mary's Hospital, College of Medicine, The Catholic University of Korea, Seoul, Republic of Korea

12 Department of Hematology and Oncology, Internal Medicine, Busan Paik Hospital, Inje University College of Medicine, Busan, Republic of Korea

13 Department of Hematology, Asan Medical Center, University of Ulsan College of Medicine, Seoul, Republic of Korea

14 Department of Hematology and Medical Oncology, Kyung Hee University College of Medicine, Seoul, Republic of Korea

15 Division of Hemato-Oncology, Department of Internal Medicine, Yeungnam University College of Medicine, Daegu, Republic of Korea

16 Department of Internal Medicine, CHA Bundang Medical Center, CHA University School of Medicine, Seongnam, Republic of Korea

17 Division of Hematology/Oncology, Department of Internal Medicine, Kosin University College of Medicine, Kosin University Gospel Hospital, Busan, Republic of Korea

18 Division of Hematology and Oncology, Department of Internal Medicine, Chungnam National University Hospital, Daejeon, Republic of Korea

19 Division of Hematology, Gachon University Gil Medical Center, Gachon University College of Medicine, Incheon, Republic of Korea

20 Department of Internal Medicine, Jeonbuk National University Medical School, Jeonju, Republic of Korea

21 Division of Hematology and Oncology, Department of Internal Medicine, Hanyang University College of Medicine, Hanyang University Seoul Hospital, Seoul, Republic of Korea

22 Korea Otsuka Pharmaceutical Co., Ltd, Seoul, Republic of Korea

23 Department of Hematology, Uijeongbu Eulji Medical Center, Uijeongbu, Republic of Korea

24 Leukemia Omics Research Institute, Uijeongbu Eulji Medical Center, Eulji University Uijeongbu Campus, 712 Dongil-ro, Uijeongbu, 11759, Korea

**Corresponding Author:** Dong-Wook Kim, M.D., Ph.D.

Uijeongbu Eulji Medical Center,

Leukemia Omics Research Institute,

Eulji University Uijeongbu Campus,

712 Dongil-ro, Uijeongbu 11759, Korea

E-mail: [dwkim@eulji.ac.kr](mailto:dwkim@eulji.ac.kr)

**Supplementary Table 1.** Response by timepoint in patients with CP-CML

| Response by timepoint | Efficacy Analysis in Patients with CP-CML  N=86 | | | | | | | |
| --- | --- | --- | --- | --- | --- | --- | --- | --- |
|  | Response by week 12 | | Response by week 18 | | Response by week 24 | | Response by week 30 | |
|  | N(%) | 95% CI | N(%) | 95% CI | N(%) | 95% CI | N(%) | 95% CI |
| Hematological Response (N=80) |  |  |  |  |  |  |  |  |
| n | 47 |  | 79 |  | 79 |  | 80 |  |
| CHR | 44 (93.6) | [82.5, 98.7] | 74 (93.7) | [85.8, 97.9] | 74 (93.7) | [85.8, 97.9] | 74 (92.5) | [84.4, 97.2] |
| Molecular Response (N=81) |  |  |  |  |  |  |  |  |
| n | 35 |  | 68 |  | 75 |  | 81 |  |
| MR1 | 31 (88.6) | [73.3, 96.8] | 57 (83.8) | [72.9, 91.6] | 64 (85.3) | [75.3, 92.4] | 70 (86.4) | [77.0, 93.0] |
| MR2 | 22 (62.9) | [44.9, 78.5] | 45 (66.2) | [53.7, 77.2] | 54 (72.0) | [60.4, 81.8] | 62 (76.5) | [65.8, 85.3] |
| MMR | 9 (25.7) | [12.5, 43.3] | 20 (29.4) | [19.0, 41.7] | 27 (36.0) | [25.2, 47.9] | 35 (43.2) | [32.2, 54.7] |
| MR4.5 | 6 (17.1) | [6.6, 33.7] | 10 (14.7) | [7.3, 25.4] | 13 (17.3) | [9.6, 27.8] | 19 (23.5) | [14.8, 34.2] |
| Response by timepoint is defined as the proportion of patients who achieved the specified response at or before each respective timepoint.  N: Number of CP-CML patients who had at least one post-treatment efficacy assessment (hematologic, molecular, or cytogenetic) following ponatinib administration.  n: Number of CP-CML patients for whom treatment response data were available by each specific timepoint (week 12, week 18, week 24, and week 30).  %: Calculated as the proportion of patients achieving the response among those included in the analysis by each timepoint (denominator).  Data collected more than 28 days after ponatinib administration were excluded from this analysis. | | | | | | | | |

**Supplementary Table 2.** Summary of serious adverse drug reactions

|  | Overall (N=148) | |
| --- | --- | --- |
|  | Incidence  n (%) | Event  [case(s)] |
| **Total** | **13 (8.8)** | **[24]** |
| **Blood and lymphatic system disorders** | **4 (2.7)** | **[7]** |
| Febrile neutropenia | 2 (1.4) | [3] |
| Neutropenia | 2 (1.4) | [2] |
| Pancytopenia | 1 (0.7) | [1] |
| Thrombocytopenia | 1 (0.7) | [1] |
| **General disorders and administration site conditions** | **4 (2.7)** | **[6]** |
| Pyrexia | 4 (2.7) | [5] |
| Pain | 1 (0.7) | [1] |
| **Investigations** | **4 (2.7)** | **[5]** |
| Neutrophil count decreased | 3 (2.0) | [3] |
| Platelet count decreased | 2 (1.4) | [2] |
| **Gastrointestinal disorders** | **1 (0.7)** | **[1]** |
| Pancreatitis acute | 1 (0.7) | [1] |
| **Musculoskeletal and connective tissue disorders** | **1 (0.7)** | **[1]** |
| Myalgia | 1 (0.7) | [1] |
| **Nervous system disorders** | **1 (0.7)** | **[1]** |
| Optic nervous | 1 (0.7) | [1] |
| **Renal and urinary disorders** | **1 (0.7)** | **[1]** |
| Acute kidney injury | 1 (0.7) | [1] |
| **Respiratory, thoracic and mediastinal disorders** | **1 (0.7)** | **[1]** |
| Dyspnea | 1 (0.7) | [1] |
| **Skin and subcutaneous tissue disorders** | **1 (0.7)** | **[1]** |
| Dermatitis exfoliative | 1 (0.7) | [1] |
| MedDRA version 25.0 |  |  |
